# Study on the driving force of seasonal changes of soil erosion in Lulang-Tongmai section of Sichuan-Tibet Highway

**DOI:** 10.1371/journal.pone.0320580

**Published:** 2025-04-24

**Authors:** Xu Tong, Zhu Kangcheng, Wu Hua, Chen Yingzhu, Zhou Jianwei, Chen Linna, Kong Yuzhong

**Affiliations:** 1 School of Engineering, Tibet University, Lhasa, China; 2 Joint Laboratory of Platrau Remote Sensing, Tibet University, Lhasa, China; 3 School of Ecology and Environment, Tibet University, Lhasa, China; 4 School of Marxism, Tibet University, Lhasa, China; ICIMOD: International Centre for Integrated Mountain Development, NEPAL

## Abstract

In the context of global climate change, the section of the Sichuan-Tibet Highway from Lulang to Tongmai has become a focal point for soil erosion research due to its unique geographical location and complex natural environment. Studies have shown that between 2000 and 2023, soil erosion intensity in this area has decreased. However, erosion intensity increases from southwest to northeast, with particularly severe erosion near the Tongmai Grand Bridge and Polong Gou Grand Bridge. Correlation analysis reveals a significant positive correlation between rainfall and soil erosion, while vegetation cover is positively correlated with erosion in the short term but contributes to reduced erosion over the long term. Geodetector analysis indicates that the main driving factors vary by season. In spring, summer, and autumn, temperature and precipitation are the primary drivers, with driving forces of 0.78 and 0.75, 0.80 and 0.78, and 0.75 and 0.73, respectively. In winter, temperature and elevation are the dominant factors, with driving forces of 0.63 and 0.42. The interaction between temperature, precipitation, and other factors significantly influences soil erosion, particularly in spring and summer, where the interaction driving force exceeds 0.75. These findings provide both theoretical support and decision-making guidance for soil erosion control along the Sichuan-Tibet Highway.

## 1. Introduction

Soil erosion, a process driven by natural forces such as hydraulic action, wind, and freeze-thaw cycles, alongside human activities, disrupts, abrades, and relocates soil and other surface materials, ultimately leading to deposition [1]. As global warming garners increasing attention, soil erosion has become a critical focus of global ecological security. Globally, severe soil erosion poses an ongoing threat to ecological balance, increasingly impacts local economies, and contributes to the impoverishment of communities [2]. Highways, as indispensable infrastructure within national economies, are crucial to societal development. However, highway construction closely interacts with natural environments [3], inevitably damaging ecosystems along its path [4]. For instance, during highway construction, soil excavation disrupts existing soil structures and alters the surface and geomorphology, creating new micro-topographies [5]. Highway construction-induced soil loss, a typical example of anthropogenically accelerated erosion [6], along with emissions of dust, gases, and wastewater, significantly impacts local air and soil quality. Additionally, highway construction devastates native vegetation, reduces biodiversity, and shrinks habitats and food sources for wildlife. Data indicate that each new kilometer of highway can occupy up to 5–7 hectares of land [7]. Enhanced soil erosion due to highway construction was observed by Hoover [8], who noted that 90% of river sediment in North Carolina’s forested areas originates from roads. Vollmer et al. [9]found that road construction significantly impacts shrub vegetation growth on sandy surfaces. Costa, Baker [10], and Lal [11] observed that extensive exposure of bare surfaces during road construction exacerbates soil erosion. Selkirk et al. [12] confirmed through observations that road construction promotes gully and gravitational erosion. Ziegler and Giambelluca [13] argue that soil erosion induced by road construction is a significant component of regional soil erosion. Jones et al. [14] discovered that road construction substantially increases soil erosion. Vinson et al. [15] evaluated the applicability of various soil erosion models based on measured erosion rates from logging roads in Virginia’s mountainous regions, finding that the USLE Forest model is most suitable for this area. In comparison, research on highway-related soil erosion in China started relatively late. Zheng Shiqing and Zhou Peihua [16] observed the erosion modulus on gullied slopes of roads in the Loess Plateau during minor rainfall events. Xiao et al.[17] explored the relationship between soil erosion and highways in Jiangxi Province, while Huang Yu [18]used field surveys and simulated rainfall experiments to study soil erosion characteristics on the spoil slopes of the Duma Expressway.

Early studies primarily utilized correlation coefficients [19] to examine the spatiotemporal relationships between soil erosion and influencing factors such as topography and climate. A subset of studies employed partial correlation coefficients [20] to assess the indirect impacts of interactions between topographic and climatic factors on soil erosion dynamics. However, these approaches lacked the capacity to quantify the specific driving forces underpinning variations in soil erosion. Over time, methodologies such as multiple linear regression [21] and residual analysis were progressively refined and widely adopted. Grey relational analysis [22], while analogous to correlation coefficients in measuring relationships, employs distinct computational algorithms. Granger causality tests [23] are designed to evaluate causal relationships but remain inadequate for inferring complex interactions among multiple variables. Regression models fail to capture the spatial heterogeneity in the contributions of various factors. Biophysical process-based models introduce substantial uncertainties due to their intricate structures and extensive parameterization. Residual analysis [24] is unable to accommodate the divergent contributions of factors influencing soil erosion across varying ecosystems. The Geographical Detector [25] provides a superior capability to disentangle the individual effects of various factors on soil erosion dynamics. Its enduring application stems from its unique ability to quantify the driving forces of diverse factors with remarkable precision.

Given the lack of research data on the Qinghai-Tibet Plateau, particularly along the Sichuan-Tibet Highway, this study focuses on the Lulang to Tongmai section. Using the Revised Universal Soil Loss Equation (RUSLE) model, we obtained soil erosion data for the study area spanning the past 24 years. The analysis of these data revealed trends in soil erosion across different seasons and years, along with their spatial distribution. The study also examined the varying effects of rainfall and vegetation cover on soil erosion correlations at seasonal and annual scales. Additionally, the Geodetector was employed to calculate the influence of key driving factors and rank them, providing insights for future soil erosion prevention efforts.

## 2. Research area

**Fig 1:**
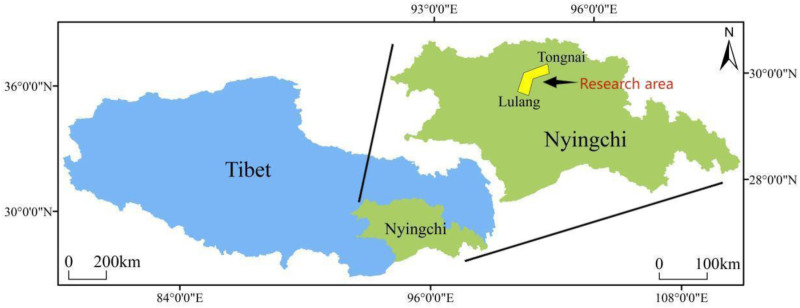
Study area transportation location map.

The Qinghai-Tibet Plateau, the highest and largest unique geographic entity globally, is renowned as the “Roof of the World” and the “Third Pole.”[26] Its extensive glacier coverage earns it the designation as Asia’s “Water Tower.” As the world’s largest plateau ecosystem, the Qinghai-Tibet Plateau is exceptionally sensitive to global climate changes [27], serving as a barometer for climatic shifts in Asia and the Northern Hemisphere. Dominated by freeze-thaw erosion due to its complex and varied geomorphology. However, with global warming, the Plateau’s ecological environment has become increasingly responsive to climatic changes, with accelerated permafrost and glacier melting, expanding water bodies, leading to evident grassland degradation and land desertification, making soil erosion a significant impediment to maintaining ecological stability and functionality [28,29]. The Lulang-Tongmai section of the Sichuan-Tibet Highway, stretching 107 kilometers from Kangge Village in Bomi County to Dewu Village in Bayi District, traverses the southeastern region of the Qinghai-Tibet Plateau, primarily served by national and county roads, with moderate transport conditions Fig 1.

The region is characterized by a highly complex topography, with an extensive network of mountain ranges and deep valleys, coupled with significant elevation differences. The geological formations exhibit considerable diversity, with fractured rocks and substantial variations in the engineering properties of the rock-soil bodies. Situated in proximity to fault zones with active crustal movements, the area experiences abundant rainfall, intense river incision, and frequent groundwater activity. Additionally, adverse geological phenomena, such as landslides, debris flows, rockfalls, and collapses, are recurrent, leading to exceptionally complex and unique soil erosion challenges in the region.

## 3. Data sources

The dataset of Table 1 was processed using ArcGIS software, with the Lulang-Tongmai section of the Sichuan-Tibet Highway serving as the vector boundary for mask clipping. Kriging interpolation was subsequently applied to standardize the spatial resolution to 30 meters.

**Table 1:** Summary of data sources.

Data name	Data sources	Spatial resolution	Time resolution
Vegetation cover data [30]	National Tibetan Plateau Data Center (http://data.tpdc.ac.cn)	250 m	Monthly 2000–2023
Rainfall data [31]	National Earth System Science Data Center (http://www.geodata.cn)	1 km	Monthly 2000–2023
Temperature data [31]		1 km	Monthly 2000–2023
DEM data		30 m	Yearly 2019
Land use resource data [32]	Chinese Academy of Sciences’ Institute of Geographic Sciences and Natural Resources Research Data Center (http://www.resdc.cn)	30 m	Yearly 2000–2023
Soil type data [33]		1 km	Yearly 1995

## 4. Research methods

### 4.1 RUSLE model

The Revised Universal Soil Loss Equation (RUSLE) [34] incorporates rainfall erosivity, vegetation cover, soil erodibility, topography (slope and slope length), and soil conservation practices as key factors to quantitatively estimate soil erosion. With relatively few parameters and easily accessible factors, RUSLE is among the most widely applied models for soil erosion estimation worldwide and has been extensively utilized in northwestern China. Map algebra refers to the process of overlaying pre-processed factor layers based on the RUSLE model. Using ArcGIS, these factor layers are multiplied through spatial operations to generate soil erosion maps for the study area.


$$Q=R\times K\times L\times S\times \left(1-C\times P\right)$$ (1)

In this equation, *Q* represents the soil erosion modulus (t·km^-2^·a^-1^), *R* denotes rainfall erosivity (MJ·mm·hm^-2^·h^-1^·a^-1^), and *K* is the soil erodibility factor (t·hm^2^·h·MJ^-1^·mm^-1^·hm^-2^). *L* and *S* are the slope length and slope steepness factors, respectively. *C* refers to the cover management factor, while *P* accounts for the effect of soil conservation practices. The factors *L*, *S*, *C*, and *P* are dimensionless.

#### (1) Rainfall erosivity factor (*R*).

The *R* factor [35] represents the contribution of rainfall to soil erosion during precipitation events.


$$R_{i}=0.2686\left[1+0.5412cos\left(\pi /6\times j-7\pi /6\right)\right]P_{d}^{1.7265}$$ (2)

In this equation, *R*_*i*_ denotes the rainfall erosivity for the ith year (MJ·mm·hm^-2^·h^-1^·a^-1^), and *P*_*d*_ represents the precipitation for the jth year (mm).

#### (2) Soil erodibility factor (*K*).

In this study, the estimation of the K factor follows the EPIC model proposed by Sharpley et al. [36] The mathematical expression is given by:


$$\begin{array}{l} K=\left\{0.2+0.3e^{\left[-0.0256W_{d}\left(1-W_{i}/100\right)\right]}\right\}\times {\left(W_{i}/\left(W_{i}+W_{t}\right)\right)}^{0.3}\times \left[1-0.25W_{c}/\left(W_{c}+e^{\left(3.72-2.95W_{c}\right)}\right)\right]\times \\ \;\left[1-0.7W_{n}/\left(W_{n}+e^{\left(-5.51-22.9W_{n}\right)}\right)\right] \end{array}$$ (3)

$$W_{n}=1-W_{d}/100$$ (4)

In the equation: *W*_*d*_ represents the sand content (%); *W*_*i*_ denotes the silt content (%); *W*_*t*_ refers to the clay content (%); and *W*_*c*_ indicates the organic carbon content (%).

#### (3) Topographic factor (*LS*).

The estimation of the *L* factor is based on a modified formula by Zhang et al.[37], which builds upon the work of McCool et al.[38] The calculation of the *S* factor follows the formula proposed by McCool et al.[39] The expression is given as:


$$L={\left(\lambda /22.13\right)}^{m}$$ (5)

$$m=\beta /\left(\beta +1\right)$$ (6)

$$\beta =\left(sin\theta /0.0896\right)/\left[3{\left(sin\theta \right)}^{0.8}+0.56\right]$$ (7)

$$S= \{\begin{array}{c} 10.8sin\theta +0.03\theta <5{}^{\circ}\\ 16.8sin\theta -0.505{}^{\circ}\ll \theta <10{}^{\circ}\\ 21.9sin\theta -0.9610{}^{\circ}\ll \theta \end{array}$$ (8)

In the equation: *λ* denotes the cumulative slope length in the horizontal direction; *m* represents the slope length exponent; *β* is a factor related to the slope gradient; and *θ* refers to the slope angle (°).

#### (4) Vegetation cover and management factor (*C*) and soil conservation practice factor (*P*).

Currently, there is no standardized method for calculating the *C* and *P* factors. In this study, these factors are assigned values based on the classification criteria provided by Feng et al.[40] The specific assignment criteria are listed in Table 2.

**Table 2:** Criteria for assigning values to C and P.

Land use type	paddy field	dryland	grassland	shrub	woodland	Other woodland	Other land
C	0.10	0.22	0.04	0.01	0.01	0.04	0.00
P	0.15	0.40	1.00	1.00	1.00	0.70	0.00

Using the Revised Universal Soil Loss Equation (RUSLE) model, we incorporated rainfall erosivity, vegetation cover, soil erodibility, topographic characteristics (including slope and slope length), and soil conservation practices as factors to quantitatively assess soil erosion. The calculation process begins by computing rainfall erosivity (*R*) using Equation 2. Soil erodibility (*K*) is then determined using Equations 3 and 4. Topographic factors (slope and slope length) are represented by the *LS* value, calculated through Equations 5, 6, 7, and 8. Additionally, the specific values for vegetation cover (*C*) and soil conservation practices (*P*) were obtained based on the standards provided in Table 2.

To comprehensively assess the characteristics of soil erosion changes in the study area, we followed the Classification and Grading Standard for Soil Erosion (SL190–2007)[41] issued by the Ministry of Water Resources of the People’s Republic of China (2007). Based on soil erosion modulus, erosion intensity is categorized into six levels: slight (< 500 t·km⁻²·a⁻¹), light (500–2500 t·km⁻²·a⁻¹), moderate (2500–5000 t·km⁻²·a⁻¹), strong (5000–8000 t·km⁻²·a⁻¹), very strong (8000–15000 t·km⁻²·a⁻¹), and severe (>15000 t·km⁻²·a⁻¹). To provide a more detailed representation of the spatial distribution of slight erosion, areas with a soil erosion modulus below 500 t·km⁻²·a⁻¹ were further divided into four sub-levels: 0–50 t·km⁻²·a⁻¹, 50–100 t·km⁻²·a⁻¹, 100–300 t·km⁻²·a⁻¹, and 300–500 t·km⁻²·a⁻¹.

### 4.2 Geodetector

Geodetector is a statistical method based on spatial statistics and spatial autocorrelation theory. It enables the exploration of spatial heterogeneity and reveals the magnitude and significance of the impact of individual driving factors on the target variable. Additionally, it identifies risk zones, assesses interaction strengths among factors, and facilitates ecological research. In this study, the factor detection and interaction detection tools within the Geodetector framework are employed to analyze the driving forces behind soil erosion changes in the study area.

(1) Factor Detection: This tool is used to detect the spatial heterogeneity of the dependent variable Y (soil erosion values) and to evaluate the extent to which independent variables X influence the spatial heterogeneity of Y. The degree of influence is represented by q [42], and the formula is as follows:

$$q=1-\left(1/N\sigma^{2}\right)\overset{L}{\underset{h=1}{\sum }}N_{h}\sigma_{h}^{2}=1-SSW/SST$$ (9)

$$SSW=\overset{L}{\underset{h=1}{\sum }}N_{h}\sigma_{h}^{2}$$ (10)

$$SST=N\sigma^{2}$$ (11)

In the equation: *h*=1, 2, ⋯, *L*, where *L* represents the categories of *X* or *Y*; the value range of *q* is [0,1]. A higher *q* value indicates a stronger impact of *X* on the spatial heterogeneity of *Y*. *N*_*h*_ and *N* represent the number of units in the *h*th category and the total number of units in the study area, respectively. *σ*_*h*_^*2*^ and *σ*^*2*^ denote the variance of *Y* within the hth category and the overall region, respectively. *SSW* and *SST* represent the sum of variances within *L* categories and the total variance of the study area, respectively.

(2) Interaction Detection: This method identifies the interaction between different independent variables *X*. It evaluates whether the joint effect of two factors on *Y* is correlated or independent. The degree of their combined impact is expressed by the *q*-value q(X1∩X2).

## 5. Results

### 5.1 Characterization of spatial and temporal variations in soil erosion

**Fig 2:**
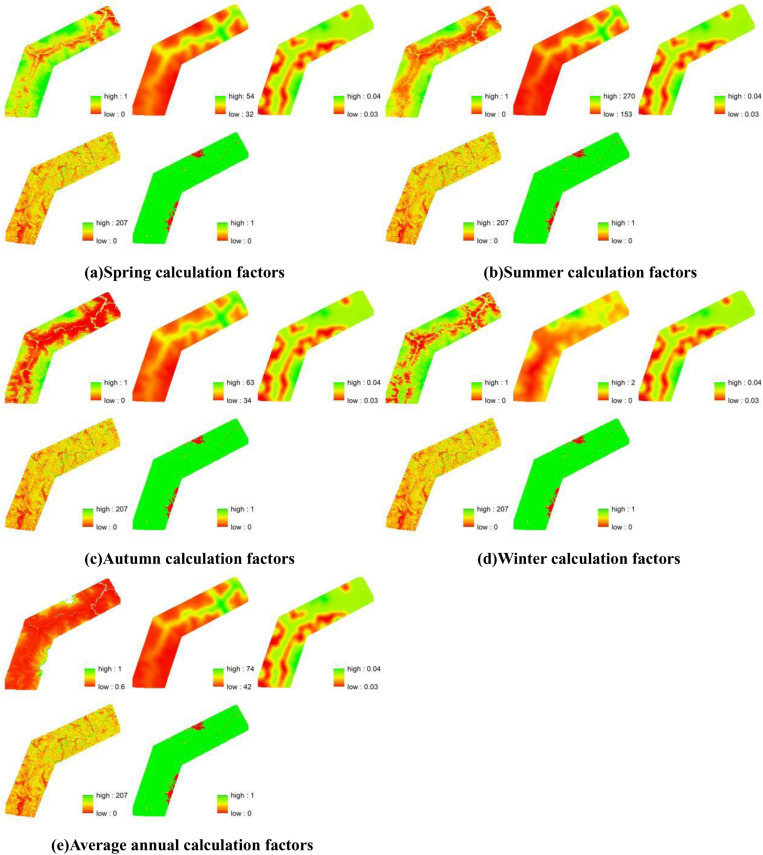
Soil erosion modeling calculation factors (from left to right, top to bottom, vegetation cover and management factor (C), rainfall erosivity (R), soil erodibility (K), topography factor (LS), and soil and water conservation measures factor (P)).

**Fig 3:**
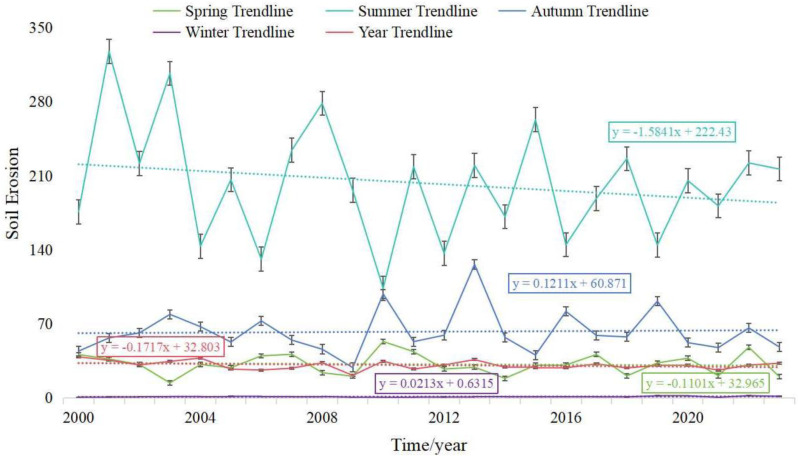
Plot of inter-annual trends in soil erosion by season.

**Fig 4:**
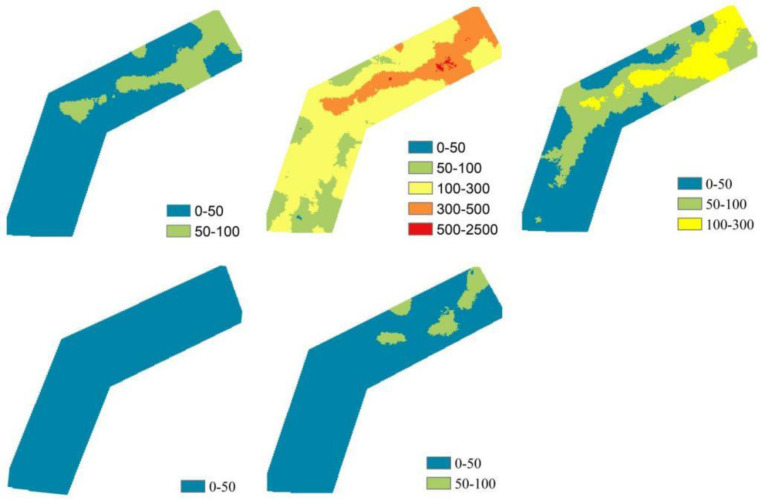
Spatial zoning map of soil erosion (from left to right, top to bottom, spring, summer, fall, winter, annual average).

Fig 2 presents the seasonal averages of the model factors over the past 24 years in the study area. Using the calculation method in Equation 1, we obtained the annual soil erosion values (*Q*) for each season from 2000 to 2023 in the study area. As shown in Fig 3, we observed that soil erosion values in the Qinghai-Tibet Plateau decreased gradually during spring from 2000 to 2023 at a rate of 0.11 t·km⁻²·a⁻¹, with a faster decline in summer, reaching -1.58 t·km⁻²·a⁻¹. In contrast, soil erosion values increased during autumn and winter at rates of 0.12 and 0.02 t·km⁻²·a⁻¹, respectively. Shown in Fig 4, reveal the distribution of average soil erosion intensity in the Qinghai-Tibet Plateau over the past 24 years. Except for summer, the average erosion intensity in all other seasons falls within the slight category. In spring, most areas in the study region exhibit soil erosion intensities within the 0–50 t·km⁻²·a⁻¹ range, with approximately 20% of the area falling between 50–100 . In summer, about 60% of the area falls within the 100–300 t·km⁻²·a⁻¹ range, with 17% and 20% of the area falling into the 50–100 t·km⁻²·a⁻¹ and 300–500 t·km⁻²·a⁻¹ categories, respectively. In autumn, the soil erosion intensity is distributed across 0–50 t·km⁻²·a⁻¹, 50–100 t·km⁻²·a⁻¹, and 100–300 t·km⁻²·a⁻¹ categories, accounting for 46%, 37%, and 17% of the area, respectively. In winter, the soil erosion intensity throughout the study area remains below 50 t·km⁻²·a⁻¹. On an annual scale, over 90% of the area exhibits soil erosion intensities within the 0–50 t·km⁻²·a⁻¹ range. Overall, the soil erosion intensity in the study area is relatively low, with a spatial trend of increasing from southwest to northeast. The soil erosion intensity between the Polong and Tongmai sections is significantly higher than in other areas, particularly near the Tongmai Grand Bridge and Polonggou Grand Bridge along the Sichuan-Tibet Highway. In summer, soil erosion intensity exceeds 500 t·km⁻²·a⁻¹, raising concerns about potential foundation settlement or deformation, which could jeopardize the structural stability of the bridges and pose risks to the safety of vehicles and passengers.

**Fig 5:**
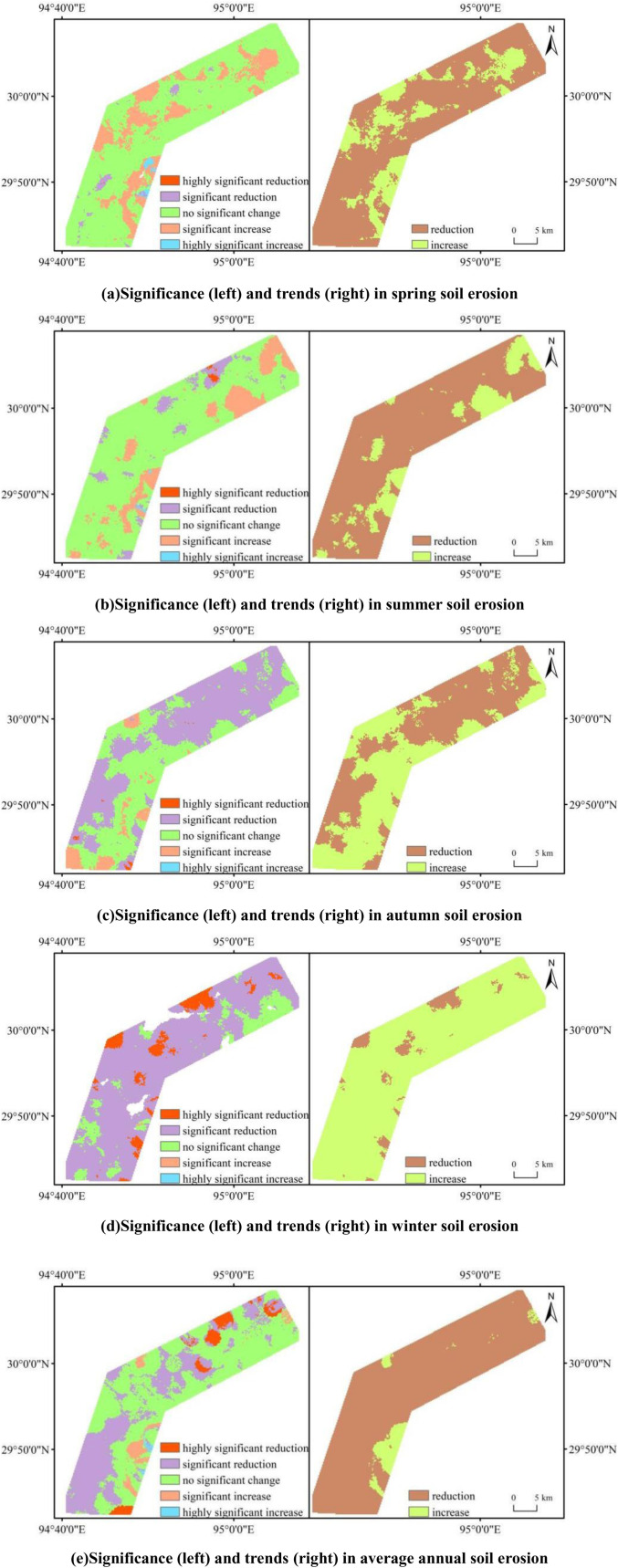
Map of soil erosion trends.

Using the Theil-Sen median trend analysis, we identified seasonal trends in soil erosion across the study area, as shown in Fig 5. In spring, soil erosion in the study area showed predominantly no significant change, covering approximately 75% of the total area. Areas with significant increases accounted for about 22%. Of the regions showing a decreasing trend, around 75% overlapped with areas of no significant change, indicating a weak significance in the decreasing trend. In contrast, the regions with increasing trends aligned with those showing significant increases, demonstrating stronger significance in the upward trend. Similarly, in summer, regions exhibiting a decreasing trend accounted for about 82% of the total area, with most of these areas showing no significant change, indicating weak significance in the decreasing trend. The remaining 18% of the area showed an increasing trend, with approximately 16% experiencing significant increases, indicating stronger significance in the upward trend. In autumn, 53% of the area exhibited a decreasing trend with weak significance, while 40% showed significant increases, indicating a predominantly significant upward trend. In winter, soil erosion trends differed markedly from other seasons, with a primarily insignificant increasing trend. The remaining 7% of the area exhibited significant increases. On an annual scale, soil erosion predominantly exhibited a decreasing trend, covering 94% of the area, with 35% showing significant decreases and 5% exhibiting highly significant decreases. Overall, except for winter, soil erosion in the study area predominantly showed a decreasing trend. Spatially, the Lulang to Zhiba section in the southwest exhibited a more pronounced decreasing trend, while other areas showed weaker significance, indicating an overall improvement in soil erosion conditions. However, the area near the Tongmai Grand Bridge consistently exhibited a significant increasing trend in soil erosion throughout the year, warranting further analysis of the influencing factors to facilitate targeted management.

### 5.2 Correlation analysis of factors affecting soil erosion

**Fig 6:**
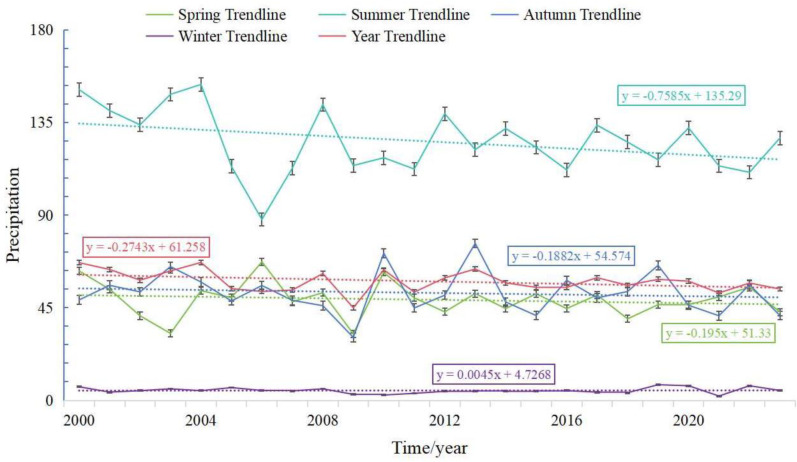
Plot of inter-annual trends in precipitation by season.

**Fig 7:**
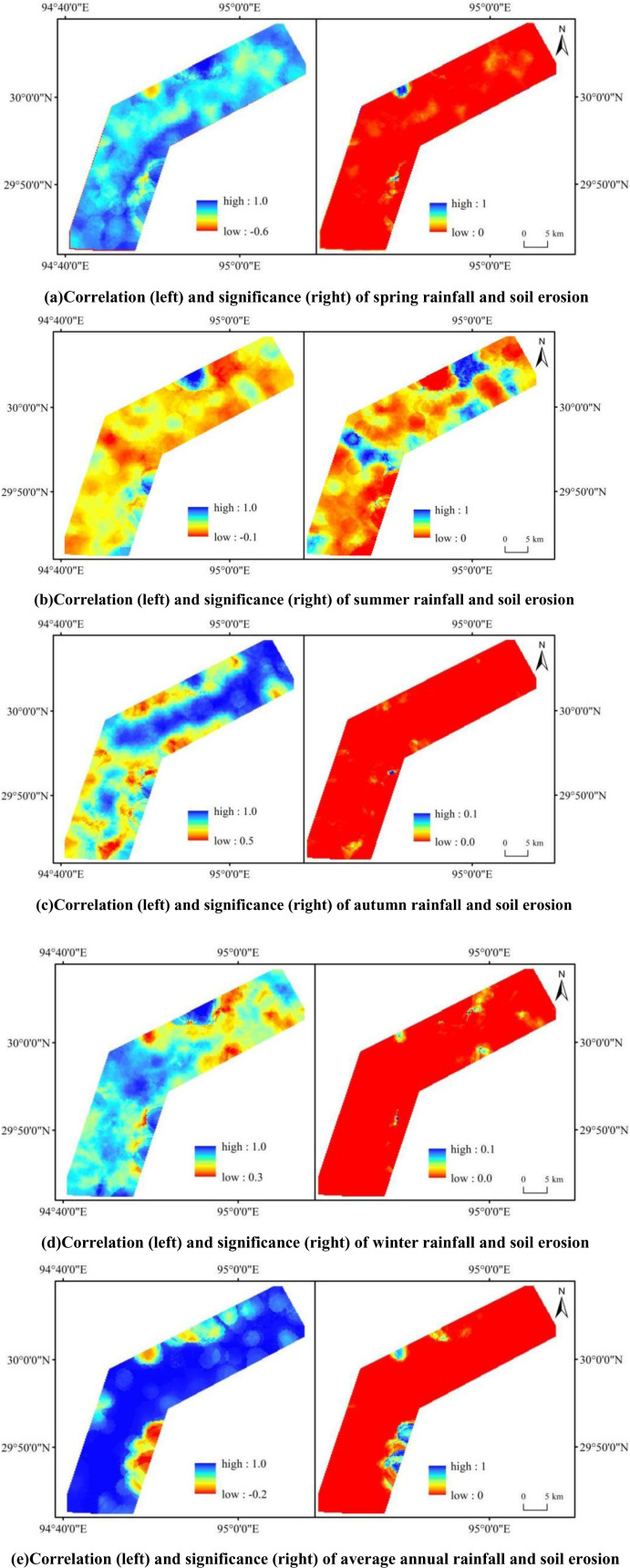
Correlation graph between rainfall and soil erosion.

The Lulang to Tongmai section of the Sichuan-Tibet Highway is located in the southeastern region of the Qinghai-Tibet Plateau, characterized by a temperate, humid plateau climate with distinct dry and rainy seasons. The rainy season occurs primarily from May to September, accounting for over 90% of the annual precipitation. In contrast, the seasonal variation in soil erodibility, topography, and soil conservation measures is relatively minor. To analyze the impact of varying rainfall levels during the dry and rainy seasons on soil erosion over the past 24 years, we first examined the annual variation in rainfall, as shown in Fig 6. Summer rainfall in the study area was significantly higher than in other seasons, followed by autumn and spring, with the lowest rainfall occurring in winter. Specifically, summer rainfall decreased at an average rate of 0.76 mm per year, while spring and autumn rainfall declined at rates of -0.20 mm·n⁻¹ and -0.19 mm·n⁻¹, respectively. In contrast, winter rainfall showed a slight increasing trend. The seasonal correlation between rainfall and soil erosion was analyzed, as shown in Fig 7. In spring, rainfall and soil erosion exhibited a significant positive correlation, covering 99% of the total area. The correlation values were distributed as follows: 0–0.2 (1%), 0.2–0.4 (6%), 0.4–0.6 (41%), and above 0.6 (51%). In summer, significant positive correlations between rainfall and soil erosion were observed in 97% of the total area. The correlation values were distributed as: 0–0.2 (31%), 0.2–0.4 (54%), 0.4–0.6 (9%), and above 0.6 (3%). In autumn, rainfall and soil erosion also exhibited significant positive correlations, with 1% of the area having correlation values between 0.4–0.6 and 99% above 0.6. In winter, significant positive correlations between rainfall and soil erosion were also found, with 1% of the area having values between 0.2–0.4, 7% between 0.4–0.6, and 92% above 0.6. On an annual scale, the results were consistent with the seasonal analysis, showing significant positive correlations, with 87% of the area having values above 0.6. Overall, the strongest positive correlation between rainfall and soil erosion was observed in autumn, followed by winter and spring, with the weakest correlation found in summer.

**Fig 8:**
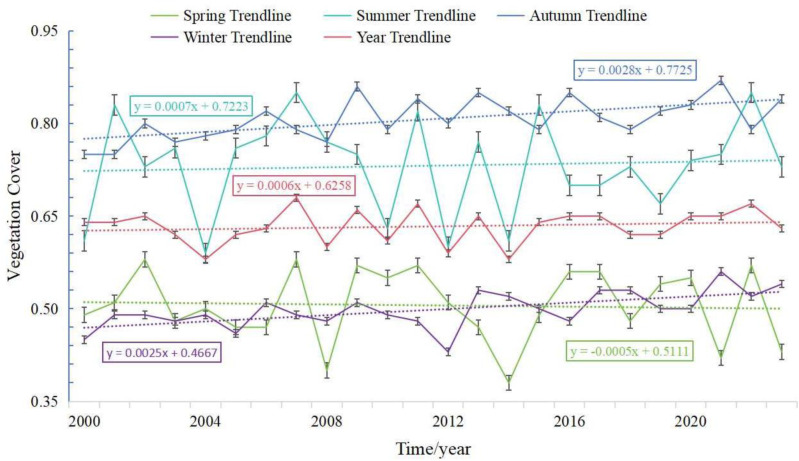
Plot of inter-annual trends in vegetation cover by season.

**Fig 9:**
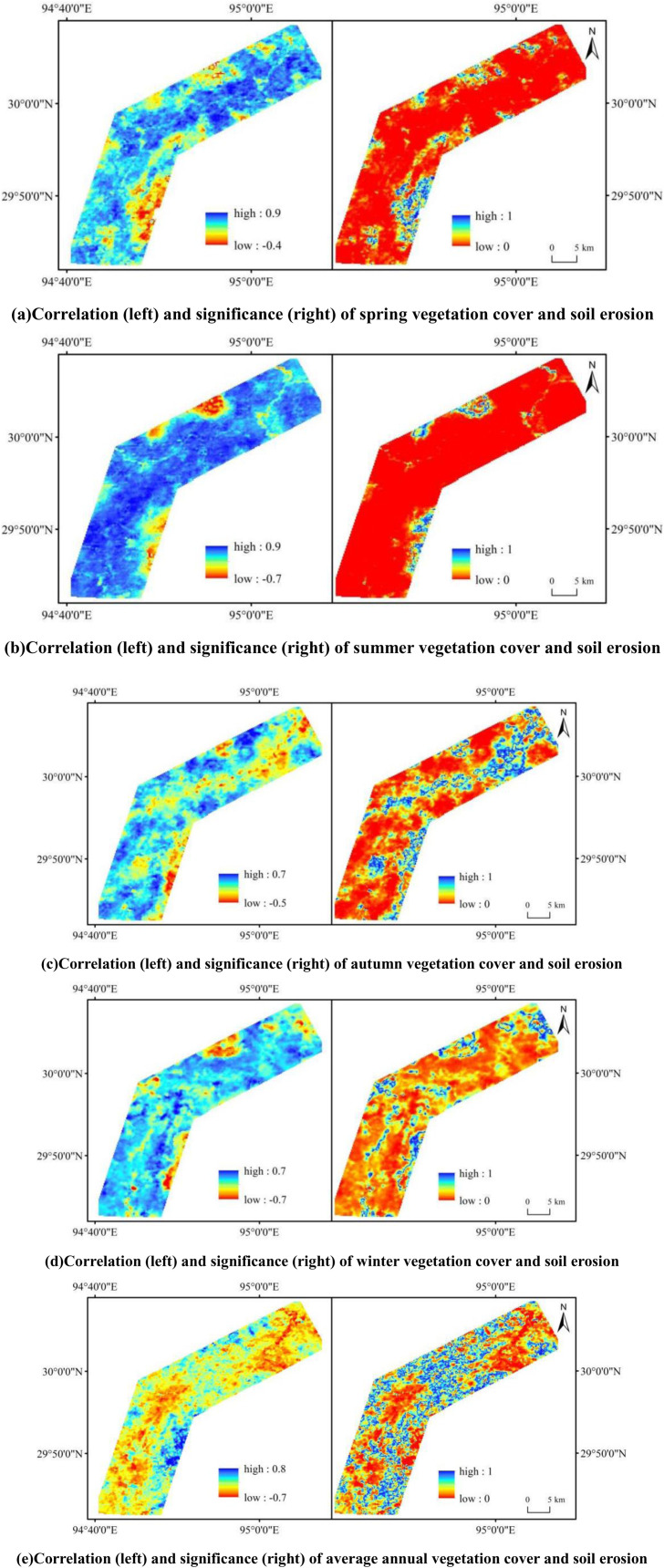
Correlation graph between vegetation cover and soil erosion.

Due to the influence of rainfall, vegetation cover exhibits significant seasonal variation. To further explore the impact of vegetation cover on soil erosion along the Lulang to Tongmai section of the Sichuan-Tibet Highway, we analyzed the temporal variation of vegetation cover in the study area, as shown in Fig 8. Since vegetation responds to rainfall with a certain lag, the abundant summer rainfall leads to significantly higher vegetation cover during summer and autumn compared to spring and winter. However, the overall variation in vegetation cover is relatively small, with growth rates in all seasons remaining below 0.01. According to the correlation analysis between vegetation cover and soil erosion shown in Fig 9, over 96% of the area in spring exhibited a significant positive correlation between the two. The distribution of correlation values was as follows: 0–0.2 (9%), 0.2–0.4 (20%), 0.4–0.6 (43%), and above 0.6 (24%). In summer, significant positive correlations covered over 97% of the area. The distribution of correlation values was: 0–0.2 (3%), 0.2–0.4 (7%), 0.4–0.6 (22%), and above 0.6 (65%). In autumn, 90% of the area exhibited significant positive correlations between vegetation cover and soil erosion, with the area distributed as follows: 0–0.2 (26%), 0.2–0.4 (38%), 0.4–0.6 (24%), and above 0.6 (2%). In winter, over 93% of the area showed significant positive correlations, with correlation values distributed as: 0–0.2 (20%), 0.2–0.4 (61%), 0.4–0.6 (11%), and above 0.6 (1%). On an annual scale, vegetation cover exhibited a stronger negative correlation with soil erosion, covering 73% of the area. These findings suggest that the correlation between vegetation cover and soil erosion varies significantly across temporal scales: predominantly negative at the interannual scale, but primarily positive at the seasonal scale. This finding indicates that while vegetation cover may positively correlate with soil erosion in the short term, increasing vegetation cover over the long term contributes to reducing soil erosion.

### 5.3 Driving force analysis of soil erosion drivers

**Fig 10:**
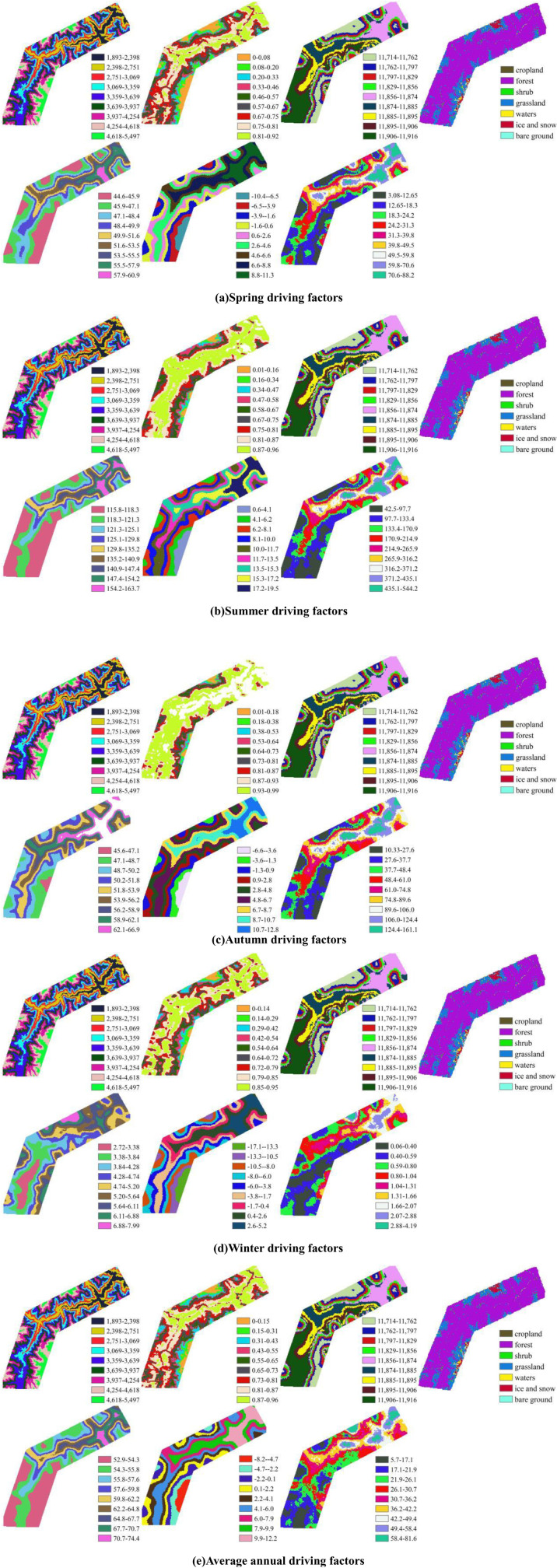
Map of soil erosion driving factors (from left to right, top to bottom, elevation (X1), vegetation cover (X2), soil type (X3), land use type (X4), precipitation (X5), temperature (X6) and soil erosion (Y)).

**Fig 11:**
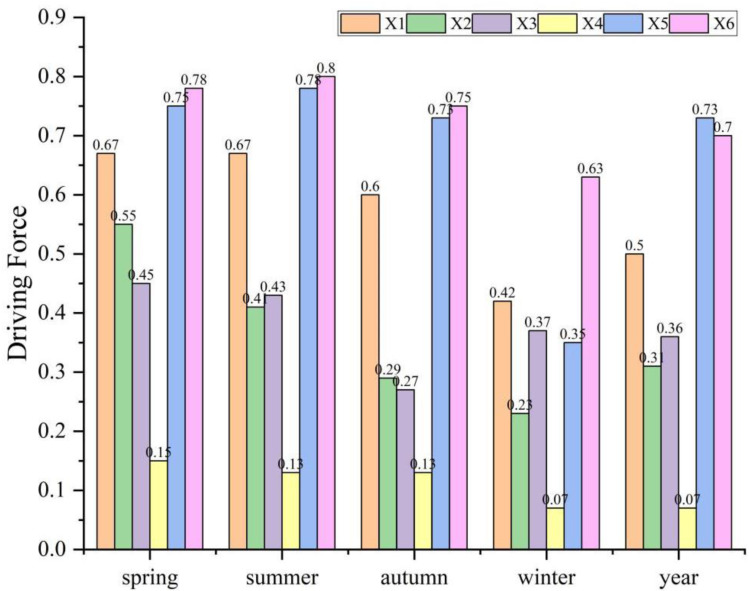
Single-factor drivers of soil erosion change (elevation (X1), vegetation cover (X2), soil type (X3), land use type (X4), precipitation (X5) and temperature (X6)).

To determine the driving forces of various factors influencing soil erosion, we first applied the natural breaks method to classify land use types into 7 categories, with other factors divided into 9 categories. The classification results are shown in Fig 10. The driving factors included in the analysis are elevation (X1), vegetation cover (X2), soil type (X3), land use type (X4), precipitation (X5), and temperature (X6). The analysis was performed by examining the univariate q-values of each driving factor to identify the dominant drivers of soil erosion (Y). Based on Geodetector, we calculated the driving forces of each factor across different seasons in the study area. The results are presented in Fig 11. The results indicate that in spring, the ranking of driving forces for each factor is as follows: temperature (0.78)> precipitation (0.75)> elevation (0.67)> vegetation cover (0.55)> soil type (0.45)> land use type (0.15). In summer, the driving force ranking is as follows: temperature (0.80)> precipitation (0.78)> elevation (0.67)> soil type (0.43)> vegetation cover (0.41)> land use type (0.13). In autumn, the ranking of driving forces is as follows: temperature (0.75)> precipitation (0.73)> elevation (0.60)> vegetation cover (0.29)> soil type (0.27)> land use type (0.13). In winter, the ranking of driving forces differs: temperature (0.63)> elevation (0.42)> soil type (0.37)> precipitation (0.35)> vegetation cover (0.23)> land use type (0.07). On an interannual scale, the ranking of driving forces is as follows: precipitation (0.73)> temperature (0.70)> elevation (0.50)> soil type (0.36)> vegetation cover (0.31)> land use type (0.07). Overall, the ranking of driving forces in winter significantly differs from that of other seasons. In other seasons, temperature, precipitation, and vegetation cover are the main driving factors, whereas in winter, temperature, elevation, and soil type have a more significant impact on soil erosion. This phenomenon is hypothesized to be closely related to the significantly reduced precipitation and vegetation cover in the study area during winter. On an interannual scale, precipitation, temperature, and elevation are the primary factors contributing to the exacerbation of soil erosion along the Lulang to Tongmai section of the Sichuan-Tibet Highway.

**Fig 12:**
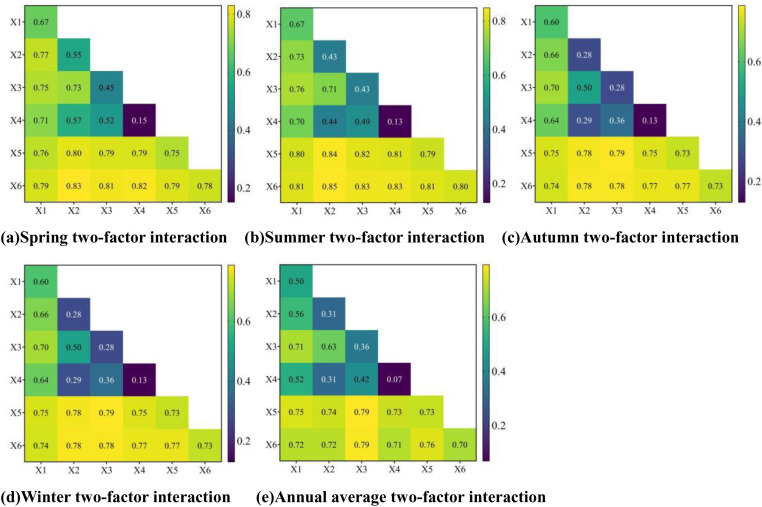
Two-factor interaction drivers of soil erosion (elevation (X1), vegetation cover (X2), soil type (X3), land use type (X4), precipitation (X5) and temperature (X6)).

To investigate the interactions of various factors on soil erosion across different seasons, we employed Geodetector to analyze the dual-factor interaction driving forces. The results are shown in Fig 12. The analysis indicates that the interaction between any two driving factors has a greater effect on soil erosion than the independent effect of each factor, primarily manifested as dual-factor enhancement and nonlinear amplification effects. Overall, the interactions between temperature, precipitation, and other factors have a significant impact on soil erosion across all seasons. Specifically, in spring and summer, the interaction between vegetation cover and temperature has the strongest driving force, with values of 0.83 and 0.85, respectively. In autumn and winter, the interaction between soil type and precipitation exhibits the highest driving force, both at 0.79. On an annual scale, the interaction between temperature and soil type has a driving force of 0.79, which is equal to that of the interaction between precipitation and soil type. These results indicate that, in high-altitude areas such as the Tibetan Plateau, the rise in temperature due to global climate change accelerates the thawing of permafrost, thereby reducing the soil’s resistance to erosion. Additionally, due to the marked seasonal distribution of precipitation in this region, abrupt changes in rainfall have a significant impact on both soil erosion and the vegetation cover’s ability to protect the soil.

## 6. Discussion

This study demonstrates that from 2000 to 2023, soil erosion along the Lulang-Tongmai segment of the Sichuan-Tibet Highway exhibited spatiotemporal patterns closely aligned with key factors, including rainfall erosivity and vegetation cover. Specifically, both peaked during summer, were moderate in spring and autumn, and reached their lowest levels in winter, mirroring the pronounced seasonal variation in soil erosion. Given that rainfall acts as the primary driver of rainfall erosivity and vegetation cover, it is hypothesized that seasonal variability in rainfall constitutes the critical factor driving changes in soil erosion. Yu Xinxiao [43] observed that rainfall intensity, represented by kinetic energy, induces splash erosion as raindrops descend from elevated heights, converting potential energy into kinetic energy upon impact with the soil surface. The magnitude of splash erosion escalates with increasing raindrop height and diameter, as these amplify the kinetic energy upon contact with the soil surface. At the interannual scale, vegetation cover exhibits a negative correlation with soil erosion, whereas at the seasonal scale, a significant positive correlation emerges, potentially linked to abrupt seasonal rainfall variations on the Qinghai-Tibet Plateau. Goebes et al. [44] and Fleischbein et al. [45] proposed that the spatial distribution of vegetation modifies raindrop kinetic energy by regulating canopy water outflow and rainfall interception, thereby impacting soil erosion processes. Goebes et al. [46] demonstrated that vegetation canopy interception effectively redistributes rainfall, thereby mitigating erosive rainfall reaching the soil surface. Seitz et al. [47] revealed that increasing vegetation height amplifies raindrop kinetic energy by a factor of 2–3, thereby exacerbating splash erosion. Spatial analysis indicates that soil erosion intensity near the Tongmai Grand Bridge and Polonggou Grand Bridge surpasses that in other areas, attributed to their positioning within the geomorphologically hazardous zones of Tongmai and Pailong. This finding underscores the significant influence of topographic factors, such as elevation and slope, on soil erosion dynamics. These results align with the findings of Wang Bingzhe et al. [48], who reported a positive correlation between elevation and soil erosion intensity in their study of soil erosion in the upper reaches of the Hutuo River in Shanxi Province. Additionally, the reduction in soil thickness during the summer and autumn tourist seasons further exacerbates soil erosion intensity. Wang Zhiqiang et al. [49] observed that in the natural grasslands of Duolun, Xilinhot, and Balinzuo Banner, Inner Mongolia, vegetation growth initially increased rapidly with soil thickness within a certain range, but beyond 20 cm, the rate of growth decelerated significantly.

However, the findings from the study on soil erosion drivers reveal that temperature consistently exerts a stronger influence on soil erosion in the study area across all seasons, surpassing both precipitation and vegetation cover. This observation contrasts with the findings of Zhang et al. [50], who suggested that high-intensity rainfall events further exacerbate soil erosion, and Zhou et al. [51], who proposed that increasing vegetation cover reduces soil erosion intensity. The Tibetan Plateau, as the highest and largest permafrost region in the middle- and low-latitude zones, has experienced rapid and significant permafrost degradation due to rising temperatures [52]. This degradation has led to a loosening of the soil structure, consequently diminishing its resistance to erosion. Moreover, the ecosystem of the Tibetan Plateau is highly fragile. In addition the reduced stability of glaciers at high altitudes may also be a cause [53,54]. Human activities, such as those associated with the Sichuan-Tibet and Qinghai-Tibet highways, directly contribute to vegetation destruction and disruption, exacerbating the downward shift of the permafrost boundary and triggering a range of issues, including freeze-thaw mudflows [55]. In contrast, the degradation of permafrost, driven by both climate change and human activities, has also severely impacted the stability of engineering activities in the Tibetan Plateau region [56,57]. While recognizing temperature as a key factor in soil erosion variation, it is equally important to consider its potential impacts on road infrastructure.The global rise in temperatures is projected to increase permafrost temperatures, transforming low-temperature permafrost into high-temperature permafrost, which is more prone to degradation and associated subsidence deformation. Research indicates that the deformation of permafrost subgrade surfaces is closely linked to temperature conditions [58], with the state and variation of permafrost subgrade temperatures determining the deformation characteristics of the subgrade [59].

This study has several limitations, such as the difficulty in quantifying the impact of engineering measures and incorporating them into the model calculations. In the future, more scientifically rigorous parameterization methods could enhance the accuracy of soil erosion estimations. Additionally, due to the limitations of data temporal resolution, this study did not utilize daily rainfall data to calculate rainfall erosivity. Future studies are encouraged to use higher-resolution data to improve the accuracy of the results. Moreover, due to data availability and the study’s spatial scale, this research focused solely on vegetation cover, despite the numerous vegetation-related factors that may influence soil erosion. Future research should consider additional influencing factors to further elucidate the mechanisms of soil erosion along the Sichuan-Tibet Highway.

## 7. Conclusion

(1) Between 2000 and 2023, significant seasonal variations in soil erosion were observed across the Tibetan Plateau, with severe erosion occurring during the rainy season (May to September) and minimal erosion during the winter months. Overall, soil erosion in the study area exhibited a decreasing trend, excluding the winter season. The intensity of soil erosion is generally mild but increases from the southwest to the northeast, with the most pronounced erosion problems observed near the Tongmai Grand Bridge and the Polong Gou Grand Bridge along the Sichuan-Tibet Highway. It is recommended that government authorities enhance soil erosion prevention measures for infrastructure, including roads and bridges, in these regions.
(2) Seasonal rainfall is significantly positively correlated with soil erosion, with the strongest correlation observed in autumn (99% of the region showing a significant positive correlation), followed by a weaker correlation in summer. Vegetation cover is significantly positively correlated with soil erosion on a seasonal scale, but negatively correlated on an annual scale. This suggests that long-term increases in vegetation cover can help reduce soil erosion, thus mitigating local soil erosion and water loss issues.
(3) Temperature and precipitation are the primary driving factors influencing soil erosion, with driving forces of 0.78 and 0.75 in spring, 0.80 and 0.78 in summer, and 0.75 and 0.73 in autumn. In winter, the driving forces of temperature and elevation are higher, at 0.63 and 0.42, which is associated with lower precipitation and reduced vegetation cover. The interaction between temperature, precipitation, and other factors significantly affects soil erosion, especially in spring and summer, where the interaction between temperature and vegetation cover exhibits a driving force of 0.83 and 0.85, and the interaction between precipitation and vegetation cover reaches 0.80 and 0.84. It is recommended that the government increase monitoring of soil erosion during these periods.

## Supporting information

S1 Data (XLSX)

S1 Text (DOCX)
