# Supplementary material for: Study on the driving force of seasonal changes of soil erosion in Lulang-Tongmai section of Sichuan-Tibet Highway
Source: PLoS One. 2025 Apr 24;20(4):e0320580. doi: 10.1371/journal.pone.0320580 (PMC12021291; doi:10.1371/journal.pone.0320580)
Supplement: S1 Text — (DOCX) [file pone.0320580.s002.docx]

**Data sources**

The dataset of Table 1 was processed using ArcGIS software, with the Lulang-Tongmai section of the Sichuan-Tibet Highway serving as the vector boundary for mask clipping. Kriging interpolation was subsequently applied to standardize the spatial resolution to 30 meters.

**Table 1. Summary of data sources**

| Data name | Data sources | Spatial resolution | Time resolution |
| --- | --- | --- | --- |
| Vegetation cover data^[1]^ | National Tibetan Plateau Data Center (http://data.tpdc.ac.cn) | 250 m | Monthly 2000-2023 |
| Rainfall data^[2]^ | National Earth System Science Data Center (http://www.geodata.cn) | 1 km | Monthly 2000-2023 |
| Temperature data^[2]^ |  | 1 km | Monthly 2000-2023 |
| DEM data |  | 30 m | Yearly 2019 |
| Land use resource data^[3]^ | Chinese Academy of Sciences' Institute of Geographic Sciences and Natural Resources Research Data Center (http://www.resdc.cn) | 30 m | Yearly 2000-2023 |
| Soil type data^[4]^ |  | 1 km | Yearly 1995 |

**References**

[1] Gao J, Shi Y, Zhang H, et al. China regional 250m fractional vegetation cover data set (2000-2023). National Tibetan Plateau / Third Pole Environment Data Center. 2022.

[2] Peng SZ, Ding YX, Liu WZ, et al. 1 km monthly temperature and precipitation dataset for China from 1901 to 2017. Earth System Science Data. 2019, 11, 1931-1946.

[3] Xu XL Liu JY, Zhang SW, et al. China multi-period land use remote sensing monitoring dataset (CNLUCC). Resource and Environmental Science Data Registration and Publication System.

[4] Liu F, Wu HY, Zhao YG, et al. Mapping high resolution National Soil Information Grids of China, Science Bulletin. 2021.
